# How We Spent Our Vacation

**Published:** 1874-07

**Authors:** 


### The Bistoury.

ELMIRA, N. Y., JULY, 1874.

The BISTOURY is published Quarterly, upon the 1st of April, July, October and January, at Fifty Cents a year, in advance.

### HOW WE SPENT OUR VACATION.

Like sensible people, did we. We were tired—or thought we were, which is much the same—and concluded to rest, in earnest. Our party was an odd one—odd as the traditional Dick's hat-band, composed as it was of a preacher, an ex-banker, an ex-merchant, a manager and a doctor.

Our forces collected, we started—started with tent, cooking utensils, provisions, blankets, fishing-tackle and John. John was our pride. 'Twas he who astonished us with his culinary feats, performed without the usual kitchen aids, and supplied us with marvelously cooked dishes, of the most savory character.

We went by rail, just fifty miles away, and landed at a fairy sort of spot, on the beautiful Lycoming, in the wilds of Pennsylvania. Our luggage being transferred to a boat, all of us, including John and our supplies, paddled up the creek to a charmingly located point of land where two creeks ran together, forming a quiet, placid pond below, in which the wary, nimble trout is wont to hide. 'Twas here we landed, pitched our tent between two majestic beeches, beneath whose brawny, crooked, scraggling limbs, so dense in the exuberance of their foliage, as to completely shelter us from storm and heat, we commenced our primitive mode of housekeeping. The preacher and doctor amused themselves in pitching the tent, each one trying to have his own whims gratified, but both satisfied with the result, and that their skulls escaped the necessity of trepanning, which the repeated falling of the ridge pole seemed likely to subject them to.

The ex-merchant, he, skilled in the use of the slender fly-rod, encased in rubber boots, from the tops of which a pair of shoulders but just emerged, and upon which hung the distinctive badge of the fly-fisherman, the landing net ; and, with broad-brimmed hat, gaily trimmed with leaders and artificial flies of various designs and hues, creel under his arm, cob pipe in mouth, which he removed just long enough to say, with a glance over his left shoulder, "boys, while you are getting the house in order, guess I'll capture some trout for dinner," away he went, with a merry song, a strain of which would occasionally reach our ear, the sentiment of which seemed to be that—"trout delicious caught are," the final sentence rising faintly but musically above the roar of the rapid, and seemed to rhyme with "water"—this was all we could hear, until a regular war-whoop rose above the ripple and splash of the sparkling stream, clearly indicating to our practiced ear that ex-M. had hooked "a buster." John, thinking the owner of the voice had fallen in the creek, dropped his axe and bounded like a deer to the scene of conflict, to find the merry fisherman standing upon a high bank with an immense trout struggling in the pool below, to free himself from the treacherous fly which had lured him to his death. John, fearing that the rod, which was bending from tip to butt under the fearful strain of the fish, might "break to flinders," as he expressed it, jumped from the bank, and attempted to drag the trout ashore by grasping the line, and was much astonished at the fisherman's quick but earnest remonstrance to such a proceeding. You see, John's fishing had been confined to the capture of mullets, eels and bullheads in old Virginia, and had not yet witnessed the delicate manipulations necessary to land a large and struggling trout. His fears for the safety of the rod was, under the circumstances, excusable, and his delight as great as that of the fisherman's, when the landing net had been safely applied, and revealed within its meshes as beautiful a pound trout as was ever landed by any enthusiastic follower of Sir Isaak.

By noon, our camp was in readiness for comfortable living. Bunks, made from flood-wood and covered with delightful spring beds, made from hemlock boughs, and upon which rested ticks of straw, procured from the neighboring farm house, invited us to a summer's afternoon snooze. Tables had been constructed ; a barrel sunken at the base of a tree and filled with ice, constituted our refrigerator. Stools had been manufactured from slabs, and John's fire place challenged our admiration, both in its construction and the facility with which it was managed. Ex-M. returning in due season with a good display of trout, they were soon consigned to the frying-pan, where, under John's skillful management, were converted into a well-browned, savory, seasoned morsel, which, with the boiled potatoes—with their jackets on—broiled salt pork, most delicious coffee, good bread and fresh butter, constituted a meal worthy of the kings—which we were.

After dinner, reclining in our arm chair, manufactured from a barrel, which had drifted from some habitation further up the creek, we enjoyed our pipe, laid plans for fishing excursions, listened to the jokes of the witty ones, and voted unanimously that we were as happy as mortals could be.

For the next three weeks our experiences were as varied as they were pleasant and jolly. Many fine trout were captured and eaten, many sent to our friends at home. Excursions were made to neighboring streams, returning to our camping grounds at night-fall, where the incidents of the day were graphically related around the camp fire, midst wreaths of smoke from the social pipe. At early morn, long before the sun had shown his head above the tall mountain tops by which we were surrounded, our breakfasts dispatched, were we out in search of the shy and sprightly trout. And many were the shouts that came ringing upon the morning air, and echoed from hill to dale, as the merry fisherman succeeded in enticing some golden hued beauty to his favorite fly. Fun! the word is tame as an expression of the sensations experienced by the true fisherman, who has captured a pound trout upon a gossamer line attached to a seven-ounce-rod. To strike a trout with such a tackle, and then have him dive for a root or bush, while your rod-tip meets your hand in your effort to keep him in the pool, and in response to the bending rod, he suddenly leaps into the air, shaking the crystal drops from his golden sides, and then, with a terrific sweep, shoots down the swift water of the rapids below, to the merry rattle of the reel, while every muscle in your body is exerted to give you a firm footing, and every nerve is tingling with excitement for fear the slender tackle will part, or the tender mouth of the trout tear from the hook—fun!—the gods in all their mythological pranks never attained to sport like this—it is inimitable—glorious!

Try it every day for three weeks, living the while in the open air, and varying the entertainment by beautifying your camp, building arbors, constructing settees and erecting other little comforts that will suggest themselves as their need is felt. Then, an occasional visit of friends from the city—particularly if they be ladies, and as jolly as the five who dined and supped with us one day—these be pleasures not soon to be forgotten, and benefits that leave their healthful influences with you always.

When night comes you are tired; retire to your inviting bunk, and are lulled to sleep by the musical ripple of the stream, the piping note of the frog, the plaintive song of the whip-poor-will, to awake at early dawn refreshed, and eager for the day's sports.

The pleasure, comfort and health that is to be derived from *such* a vacation is beyond computation. Others may seek it in vain at the fashionable sea-side resorts, and at a fearful expenditure of money and strength, but the mountain wilds, with its charming brooks and glorious air, is the resting place for us.

